# Fate of the distal aorta following root replacement in Marfan syndrome: a propensity score matched study

**DOI:** 10.3389/fcvm.2023.1186181

**Published:** 2023-06-28

**Authors:** Hao Liu, Suwei Chen, Congcong Luo, Yongliang Zhong, Zhiyu Qiao, Lizhong Sun, Junming Zhu

**Affiliations:** ^1^ Department of Cardiovascular Surgery, Beijing Aortic Disease Center, Beijing Anzhen Hospital, Capital Medical University, Beijing, China; ^2^ Department of Thoracic Surgery, Shanghai Ninth People's Hospital, Shanghai Jiaotong University School of Medicine, Shanghai, China

**Keywords:** Marfan syndrome, distal aorta, prophylactic surgery, Bentall procedure, propensity score matching

## Abstract

**Objective:**

The aortic root is the most frequent segment involved in Marfan syndrome. However, Marfan syndrome is a systemic hereditary connective tissue disorder, and knowledge regarding the outcomes of the native distal aorta after prophylactic aortic root surgery is limited.

**Methods:**

From April 2010 to December 2020, 226 patients with Marfan syndrome and 1,200 patients without Marfan syndrome who underwent Bentall procedures were included in this study. By propensity score matching, 134 patients were assigned to each group. Clinical manifestations and follow-up data were acquired from hospital records and telephone contact. The cumulative incidence of aortic events was estimated in Marfan and non-Marfan patients with death as a competing risk.

**Results:**

Patients with and without Marfan syndrome had similar baseline characteristics after propensity score matching. Differences in the aortic root (62.25 ± 11.96 vs. 54.03 ± 13.76, *P* < .001) and ascending aorta (37.71 ± 9.86 vs. 48.16 ± 16.01, *P* < .001) remained after matching. No difference was observed in the frequency of aortic adverse events between the two groups (10.5% vs. 4.6%, *P* = 0.106). The cumulative incidence of aortic events was not different between Marfan and non-Marfan patients (15.03% ± 4.72% vs. 4.18% ± 2.06%, *P* = 0.147). Multivariate Cox regression indicated no significant impact of Marfan syndrome on distal aortic events (HR: 1.172, 95% CI: 0.263–5.230, *P* = 0.835). Descending and abdominal aortic diameter above normal at the initial procedure were associated with the risk of distal aortic events (HR: 20.735, *P* = .003, HR: 22.981, *P* = .002, respectively).

**Conclusions:**

New-onset events of the residual aorta in patients undergoing Bentall procedures between the Marfan and non-Marfan groups were not significantly different. Distal aortic diameter above normal at initial surgery was associated with a higher risk of adverse aortic events.

## 1. Introduction

Marfan syndrome (MFS) is a systemic connective tissue disorder often caused by mutations of FBN1 (FBN1 gene, encoding fibrillin-1). The aortic root is the most commonly involved segment of the aorta in patients with MFS (1, 2). Prophylactic aortic root surgeries reduce the risk of type A aortic dissection and improve life expectancy in MFS patients (3, 4). However, with increasing survival time, descending aortic dilation or primary type B aortic dissection may occur in MFS patients after aortic root surgery (1, 5, 6). Aortic dissection and previous root replacement have been recognized as potential risk factors for lesions on the distal aorta in MFS (5, 7–11). Some recent studies have also described aortic aneurysm without dissection and peripheral arterial aneurysms in MFS (12–14). However, these previous studies have mostly been limited to MFS populations, and no comparisons were made between MFS and non-MFS patients. Thus, the role of MFS as an intrinsic factor in the fate of the distal aorta is not well understood.

In the present study, we sought to compare distal aortic events after the Bentall procedure between controls and patients with MFS. To better assess the influence of MFS as a single factor, propensity score matching was used to adjust for other confounders.

## 2. Materials and methods

### 2.1. Patient selection

A total of 4,054 patients who underwent the Bentall procedure at Beijing Anzhen Hospital from April 2010 to December 2020 were reviewed. We aimed to compare adverse events in the normal aorta after the Bentall procedure between the MFS and non-MFS groups. Therefore, patients with aortic root aneurysms alone were the main study population. Preexisting or concomitant distal aortic lesions were excluded, and only patients aged ≥18 years were included in this study. The detailed exclusion criteria included the following: (1) missing values in MFS diagnosis, (2) underage patients, (3) history of cardiovascular surgery, (4) acute aortic syndromes: aortic dissection, intramural haematoma and penetrating atherosclerotic ulcer, (5) pseudoaneurysm, (6) congenital aortic disease: aortic coarctation, transposition of the great arteries, (7) inflammatory or immune-related disease: infective endocarditis, Behçet disease, Takayasu arteritis, (8) combined distal aortic lesions during this time with dilation (>40 mm) of the aortic arch, descending aorta and abdominal aorta and (9) aortic stenosis. We excluded aortic stenosis for two reasons: first, almost none of the patients in the MFS group had aortic stenosis but did have aortic regurgitation; and second, the aortic wall pathology is different in patients with aortic stenosis and patients with aortic regurgitation (15, 16). Bicuspid aortic valve (BAV) was included in this study because MFS patients also have a BAV (17, 18), and BAV patients comprised a great proportion of the adult population in the non-MFS group, particularly young patients.

**Figure 1:**
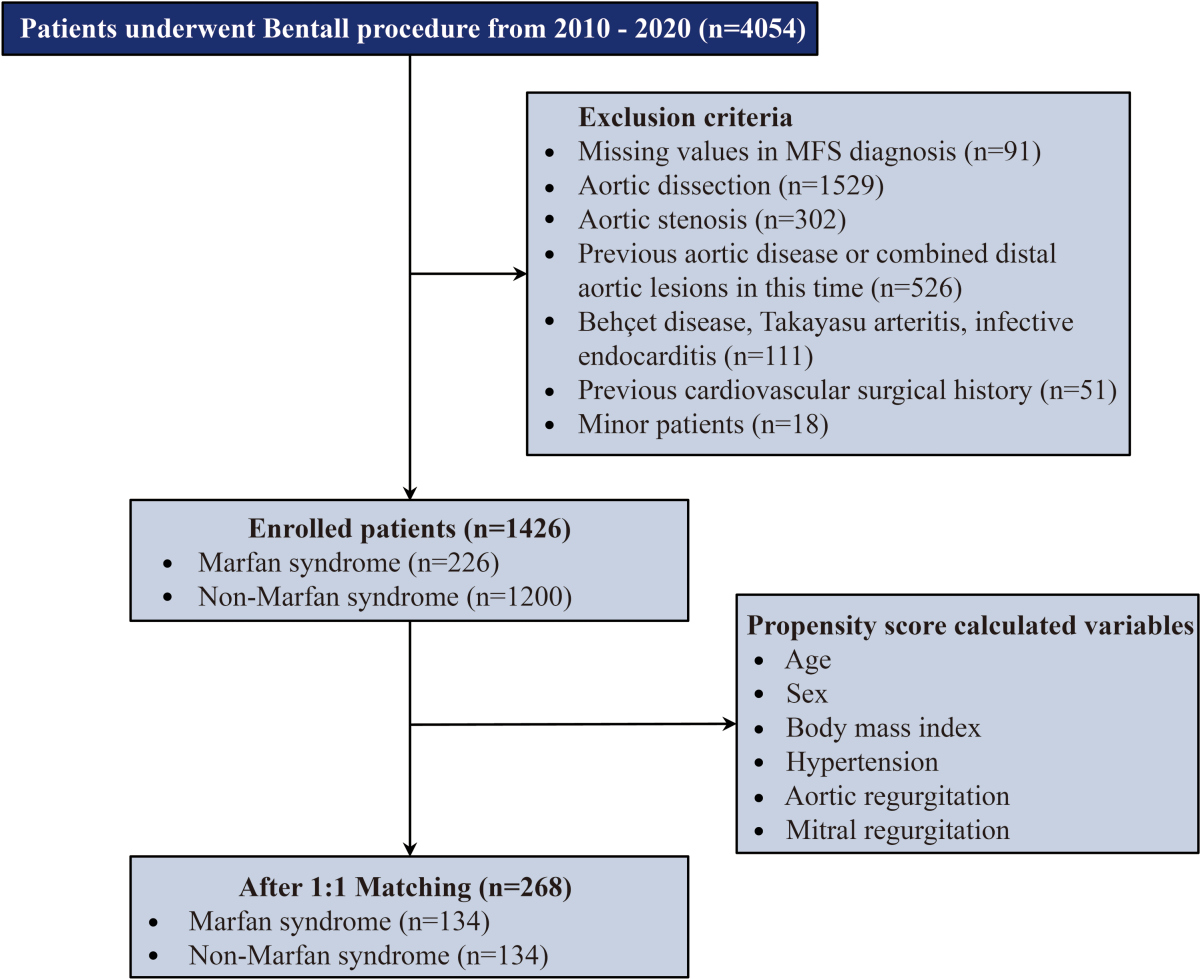
Summary flow chart of patient inclusion. Patients were matched in a 1:1 ratio with 134 patients in each group. MFS, Marfan syndrome.

Finally, 1,426 patients met the inclusion criteria. Of these, 226 patients were diagnosed with MFS, as confirmed by the revised Ghent nosology (19). A total of 134 patients were identified in both the MFS and non-MFS groups after propensity score matching for further comparison. The inclusion criteria and patient flow are displayed in Figure 1.

### 2.2. Surgical procedure

Surgeries were performed through median sternotomy with systemic heparization. All procedures utilized cold crystalloid or blood cardioplegia and standard cardiopulmonary bypass. Most of the Bentall procedures were performed with mechanical composite valve grafts. After excision of the aortic valve and completion of the suture lines between the aortic annulus and the sewing ring of the prosthetic valve, the buttons of the graft opposite the coronary ostia were excised with cautery. The aortic wall immediately adjacent to the coronary ostia was sutured to these openings with continuous 5–0 polypropylene sutures. After completion of the coronary anastomoses, the tube graft was cut to the appropriate length, and the aorta was completely transected. The tube graft was sewn to the aorta proximal to the brachiocephalic vessels. For better haemostasis, we prefer creating a Cabrol fistula from the perigraft space to the right atrium so that any postoperative bleeding will not be problematic. The remainder of the procedure, including securing haemostasis and sternal closure, was performed in a routine fashion.

### 2.3. Data collection and follow-up

The collection of demographic variables and perioperative data was performed using the medical record system. Missing data were not imputed, and patients with missing MFS diagnoses were excluded listwise. Potential factors associated with aortic aneurysm include BAV, hypertension, hyperlipoidemia, etc., were included. The aorta was divided into 5 regions based on the diverse embryonic origins and anatomic locations (20): the sinus of Valsalva (second heart field), ascending aorta and aortic arch (neural crest), descending aorta (somite), and abdominal aorta (splanchnic mesoderm). The preoperative and postoperative maximum diameter of each area was measured using echocardiography and aortic computed tomography angiography (CTA). Other combined cardiac surgeries, operative variables and postoperative data were also obtained.

Two hundred and sixty-four patients in the matched cohort were followed up by telephone or outpatient visits, and 209 (79.2%) were successfully followed up. The median follow-up time was 5.0 years (interquartile range, 3.1 to 7.7 years). The number of patients who underwent the Bentall procedure per year is shown in Supplementary Figure S1.

The primary outcome was adverse aortic events, which included aortic dilation, dissection and aortic reinterventions. Aortic dilation in the distal aorta was defined as a maximal diameter greater than 40 mm as detected by CTA. The secondary outcomes included overall deaths and other cardiovascular-related adverse events and reinterventions.

### 2.4. Statistical analysis

Continuous variables are presented as the mean ± standard deviation or median (interquartile range), and Student's t test or Mann‒Whitney *U* test was used for comparisons depending on whether the data were normally distributed. The Kolmogorov‒Smirnov test, histograms and QQ plots were used to test for normal distribution. Categorical variables were expressed as frequencies and proportions and compared by Pearson's chi-square test or Fisher's exact test.

To adjust for confounding factors between the MFS and non-MFS groups, propensity score matching was performed. Potential confounding variables (age, sex, BMI, mitral and aortic valve involvement and hypertension) were considered in a logistic regression model to generate propensity scores. Based on a calliper definition of 0.05, a 1:1 match was achieved using nearest-neighbour matching without replacement. The distributions of the propensity scores before and after matching are shown by histograms. Standardized mean differences (SMDs) were used to evaluate the match's adequacy. We used Kaplan‒Meier curves to compare the overall survival of the MFS and non-MFS groups with the log-rank test. The cumulative incidence of aortic events was calculated using the Fine and Grey regression model with death as a competing risk. A univariate Cox proportional risk model was used to select risk factors for adverse aortic events, and variables with *P *< 0.1 and MFS were used for further multivariate Cox regression analysis. The results are expressed as the hazard ratio (HR) and 95% confidence intervals (95% CI).

Statistical analyses in this study were performed using Stata/SE 15.1 for Mac (StataCorp LP, College Station, Tex), Prism 9 for Mac (GraphPad, La Jolla, Calif) and R v3.6.3 (www.r-project.org). Propensity score matching was accomplished using the package Matchit in R. A competing-risk regression model was performed using stcrreg in Stata and stcurve for plotting the cumulative incidence function.

## 3. Results

### 3.1. Patient characteristics

In the whole cohort, 226 (15.8%) patients had MFS. The MFS and non-MFS groups differed significantly in age (35.17 ± 10.02 vs. 54.41 ± 10.60, *P *< .001), BMI (21.48 ± 3.68 vs. 24.68 ± 3.60, *P *< .001) and male sex (73.9% vs. 86.0%, *P *< .001), respectively. Patients with BAV comprised 19.0% of the non-MFS group and 1.8% of the MFS group. Patients with MFS were more likely to have mitral regurgitation (27.4% vs. 16.6%, *P *< .001). The Z scores (adjusted for age, sex and body size) of the aortic root in the MFS group were also higher than those in the non-MFS group (11.47 ± 4.82 vs. 7.01 ± 4.26, *P *< .001). The prevalence of morbidities associated with age, such as hypertension, hyperlipidaemia, diabetes, stroke, and arrhythmia, was higher in non-MFS patients (Table 1).

**Table 1:** Main clinical characteristics and perioperative data of the patients.

Variables	Unmatched group				Matched group			
	MFS (*n* = 226)	Non-MFS (*n* = 1,200)	|SMD|	*P* value	MFS (*n* = 134)	Non-MFS (*n* = 134)	|SMD|	*P* value
Age, y	35.17 ± 10.02	54.41 ± 10.60	1.865	<.001	39.29 ± 10.13	39.75 ± 10.66	0.045	0.716
Male sex	167 (73.9)	1,032 (86.0)	0.305	<.001	101 (75.4)	100 (74.6)	0.017	0.888
Body mass index, kg/m^2^	21.48 ± 3.68	24.68 ± 3.60	0.882	<.001	22.79 ± 3.46	22.87 ± 3.14	0.025	0.838
Family history of aortic disease	62 (27.4)	2 (0.2)	0.859	<.001	40 (29.9)	0 (0.0)	0.919	<.001
Lens subluxation	27 (11.9)	0 (0.0)	0.520	<.001	18 (13.4)	0 (0.0)	0.555	<.001
Skeletal involvement^‡^	92 (40.7)	3 (0.3)	1.156	<.001	40 (29.9)	1 (0.7)	0.881	<.001
Bicuspid aortic valve	4 (1.8)	228 (19.0)	0.588	<.001	0 (0.0)	34 (25.4)	0.985	<.001
Hypertension	31 (13.7)	680 (56.7)	1.006	<.001	29 (21.6)	26 (19.4)	0.055	0.650
Mitral regurgitation	62 (27.4)	199 (16.6)	0.264	<.001	33 (24.6)	28 (20.9)	0.089	0.466
Aortic regurgitation	182 (80.5)	1,121 (93.4)	0.389	<.001	115 (85.8)	117 (87.3)	0.044	0.720
Diabetes	1 (0.4)	77 (6.4)	0.333	<.001	1 (0.7)	3 (2.2)	0.315	0.622
Hyperlipidaemia	3 (1.3)	76 (6.3)	0.263	0.001	2 (1.5)	5 (3.7)	0.252	0.447
Hyperuricaemia	2 (0.9)	19 (1.6)	0.063	0.559	1 (0.7)	2 (1.5)	0.563	>.999
CKD	0 (0.0)	10 (0.8)	0.130	0.379	0 (0.0)	0 (0.0)	NA†	>.999
CHD	0 (0.0)	222 (18.5)	0.674	<.001	0 (0.0)	5 (3.7)	0.024	0.060
Stroke	2 (0.9)	47 (3.9)	0.199	0.016	2 (1.5)	1 (0.7)	0.563	>.999
Arrhythmia	3 (1.3)	91 (7.6)	0.307	<.001	1 (0.7)	2 (1.5)	0.563	>.999
Z score*	11.47 ± 4.82	7.01 ± 4.26	0.980	<.001	11.06 ± 4.49	8.19 ± 5.33	0.583	<.001

Values are presented as the mean ± standard deviation or *n* (%). MFS, Marfan syndrome; SMD, standardized mean difference; CKD, chronic kidney disease; CHD, coronary heart disease.

* *Z* score, aortic root z score was measured by sinuses of Valsalva and body surface area (BSA). BSA was calculated using the Dubois and Dubois method.

^†^ NA, SMD not available for this variable.

^‡^ Skeletal involvement included wrist and thumb sign, pectus carinatum deformity, hindfoot deformity and scoliosis or thoracolumbar kyphosis.

Two hundred sixty-eight patients were identified after propensity score matching (Table 1). The distributions of the propensity scores were similar between the two groups after matching (Supplementary Figure S2). SMD <0.1 is considered to represent a negligible difference (Supplementary Figure S3). However, despite the reduction in confounding factors, the SMD of some variables remained above 0.1, and the difference persisted. The Z scores in the MFS group remained higher than those in the non-MFS group (11.06 ± 4.49 vs. 8.19 ± 5.33, *P *< .001), and the proportion of BAV in the non-MFS group increased (25.4%) after matching.

### 3.2. Characterization of patients' aorta segments

We divided the aorta into 5 parts and recorded the maximum diameter of each region preoperatively and postoperatively (Table 2). Dilation of the aorta was more frequently discovered in the aortic root (62.52 ± 12.88 vs. 52.85 ± 11.10, *P *< .001) than in the ascending aorta (36.80 ± 10.19 vs. 46.33 ± 10.05, *P *< .001) in the MFS group than in the non-MFS group. The typical pear-shaped aortic root in MFS patients persisted (root, 62.25 ± 11.96 vs. 54.03 ± 13.76, *P *< .001; ascending, 37.71 ± 9.86 vs. 48.16 ± 16.01, *P *< .001) after matching. The postoperative aortic arch and descending aorta diameters were higher in the non-MFS group before matching, and the difference was not statistically significant (*P *= 0.174 and *P *= 0.586, respectively) after matching. The abdominal aorta diameter in the non-MFS group was slightly higher than that in the MFS group (21.59 ± 3.58 vs. 22.97 ± 3.85, *P *= .002). After matching, however, MFS patients exhibited a larger abdominal aorta than non-MFS patients (22.37 ± 3.48 vs. 20.38 ± 4.03, *P *= .011). The diameters of the aortic root and ascending aorta (artificial vessel and distal residual ascending aorta) were similar in the MFS and non-MFS groups after surgery.

**Table 2:** Maximal aortic diameter at different segments.

	Unmatched group				Matched group			
Variables	MFS (*n* = 226)	Non-MFS (*n* = 1,200)	|SMD|	*P* value	MFS (*n* = 134)	Non-MFS (*n* = 134)	|SMD|	*P* value
Preoperation aorta, mm								
Root*	62.52 ± 12.88	52.85 ± 11.10	0.805	<.001	62.25 ± 11.96	54.03 ± 13.76	0.638	<.001
Ascending	36.80 ± 10.19	46.33 ± 10.05	0.941	<.001	37.71 ± 9.86	48.16 ± 16.01	0.786	<.001
Arch	26.69 ± 4.63	29.99 ± 5.08	0.677	<.001	27.55 ± 4.41	29.07 ± 5.38	0.309	0.070
Descending	22.52 ± 4.38	25.30 ± 4.14	0.650	<.001	22.92 ± 4.11	23.42 ± 4.69	0.112	0.486
Abdominal	21.32 ± 3.86	22.78 ± 3.70	0.386	0.002	22.26 ± 3.83	19.80 ± 3.51	0.671	0.005
Postoperation aorta, mm								
Root*	28.31 ± 5.36	28.75 ± 4.82	0.088	0.307	28.26 ± 5.60	28.75 ± 4.81	0.093	0.547
Artificial^†^	26.32 ± 2.60	26.56 ± 2.41	0.097	0.207	26.39 ± 2.70	26.18 ± 2.11	0.088	0.513
Ascending^‡^	29.84 ± 7.10	28.68 ± 4.64	0.194	0.437	27.76 ± 5.06	28.66 ± 4.20	0.194	0.705
Arch	26.63 ± 4.47	29.77 ± 5.11	0.655	<.001	27.45 ± 4.20	28.52 ± 5.37	0.223	0.174
Descending	22.50 ± 4.02	25.25 ± 4.13	0.674	<.001	22.90 ± 3.87	23.26 ± 4.73	0.084	0.586
Abdominal	21.59 ± 3.58	22.97 ± 3.85	0.369	0.002	22.37 ± 3.48	20.38 ± 4.03	0.527	0.011

Values are presented as the mean ± standard deviation. MFS, Marfan syndrome; SMD, standardized mean difference.

* Root represents sinuses of Valsalva (aortic root).

^†^ Artificial represents the artificial vessel in the proximal ascending aorta location.

^‡^ Ascending in the postoperation part represents the distal ascending aorta.

### 3.3. Operative data and postoperative outcomes

MFS patients had a higher rate of concomitant mitral valve surgeries (27.0% vs. 14.0%, *P *< .001) and a lower percentage of combined coronary artery bypass grafting (CABG, 2.2% vs. 12.8%, *P *< .001) than non-MFS patients. This difference was no longer significant in the matched groups. No significant differences were found between the two matched groups in terms of in-hospital death, duration of cardiopulmonary bypass, aortic cross-clamp, mechanical ventilation, ICU stay, and hospital stay (Supplementary Table S1).

### 3.4. Outcomes and reintervention of the distal aorta

**Figure 2:**
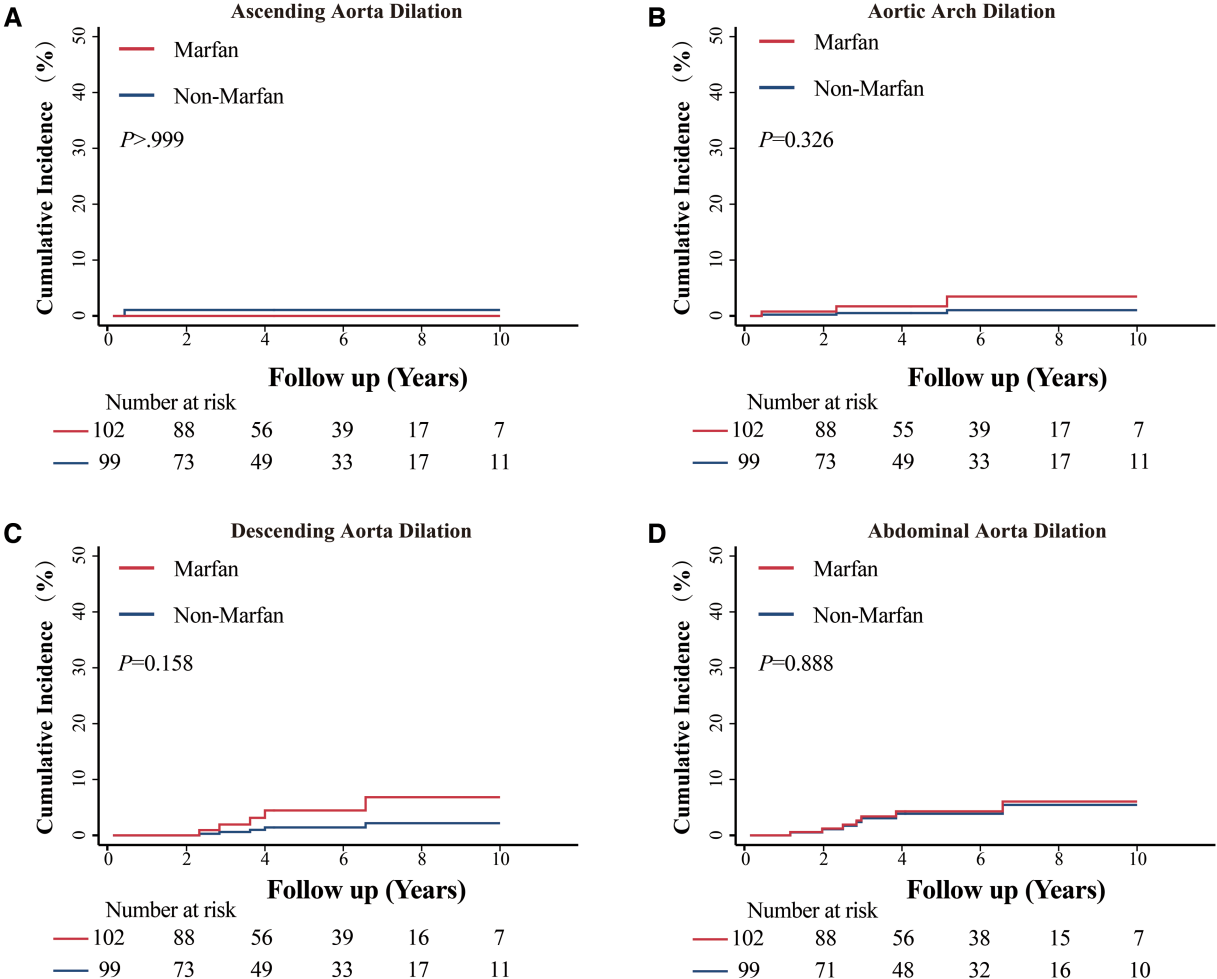
Cumulative incidence function of MFS on aortic dilation in different segments. (**A**) Comparison of the cumulative incidence of dilation in the ascending aorta between patients with Marfan syndrome (MFS) and patients without MFS. (**B**) Comparison of the cumulative incidence of dilation in the aortic arch between patients with and without MFS. (**C**) Comparison of the cumulative incidence of dilation in the descending aorta between patients with MFS and patients without MFS. (**D**) Comparison of the cumulative incidence of dilation in the abdominal aorta between patients with MFS and patients without MFS.

**Figure 3:**
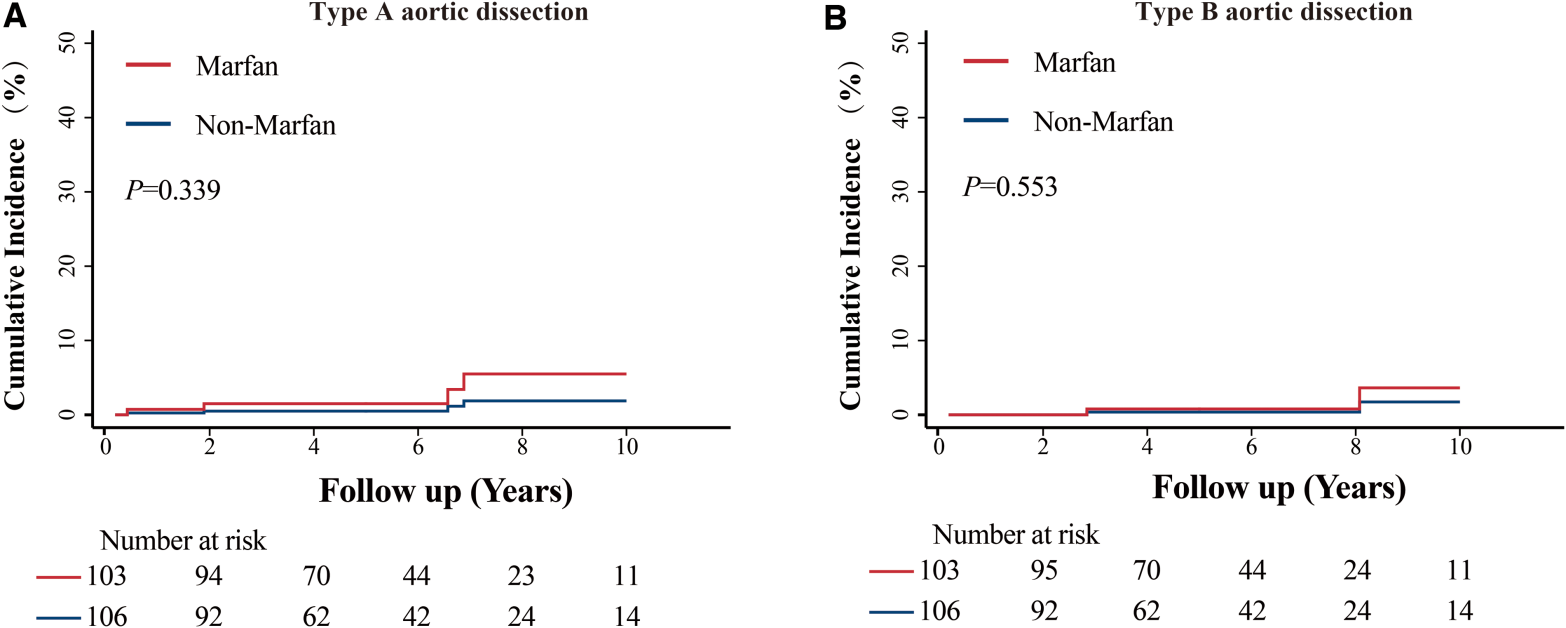
Cumulative incidence function of MFS in aortic dissection. (**A**) Comparison of the cumulative incidence of type A aortic dissection between patients with Marfan syndrome (MFS) and patients without MFS. (**B**) Comparison of the cumulative incidence of type B aortic dissection between patients with MFS and patients without MFS.

The median follow-up time was 5 years, and the interquartile range was 3.1 to 7.7 years. A total of 27 residual aortic events occurred in 11 MFS patients and 5 non-MFS patients. The incidence of adverse aortic events between the two groups was not significantly different (10.5% vs. 4.6%, *P *= 0.106). Detailed comparisons are presented in Table 3. The cumulative incidence function of the effect of MFS on the dilation of different portions of the aorta and aortic dissection showed no significant differences (Figure 2 and Figure 3). At the 10-year follow-up time point, the cumulative incidence of aortic events was 15.03% ± 4.72% in the MFS group and 4.18% ± 2.06% in the non-MFS group (*P *= 0.147) (Supplementary Figure S4). Univariate Cox regression showed possible positive correlated factors such as MFS, BMI, postoperative descending and abdominal aortic diameter on adverse aortic events (Supplementary Table S3). Descending (HR: 7.804, *P *= .018) or abdominal (HR: 16.503, *P *= .002) aortic diameter above normal (30 mm) remained statistically significant after adjustment in the multivariate Cox analysis (Table 4).

**Table 3:** Adverse events during follow-up in the matched groups.

Adverse events	MFS (*n* = 105)	Non-MFS (*n* = 108)	*P* value
All aortic-related complications	11 (10.5)	5 (4.6)	0.106
Ascending aorta dilation	0 (0.0)	1 (0.9)	>.999
Aortic arch dilation	3 (2.9)	1 (0.9)	0.364
Descending thoracic aorta dilation	5 (4.8)	2 (1.9)	0.275
Abdominal aorta dilation	4 (3.8)	3 (2.8)	0.719
Type A aortic dissection	2 (1.9)	1 (0.9)	0.618
Type B aortic dissection	2 (1.9)	1 (0.9)	0.618
Thoracoabdominal aortic aneurysm	1 (1.0)	1 (0.9)	>.999
Other cardiovascular complications			
Mitral valve regurgitation	4 (3.8)	1 (0.9)	0.208
Tricuspid valve regurgitation	2 (1.9)	0 (0.0)	0.242
Arrhythmia	5 (4.8)	5 (4.6)	>.999
Warfarin-related bleeding	2 (1.9)	0 (0.0)	0.242
Decline in heart function	4 (3.8)	0 (0.0)	0.057
Paravalvular leak	3 (2.9)	0 (0.0)	0.118
Infective endocarditis	1 (1.0)	0 (0.0)	0.493
Stroke	0 (0.0)	2 (1.9)	0.498

Values are presented in *n* (%). MFS, Marfan syndrome.

**Table 4:** Multivariate Cox regression analysis for all aortic-related adverse events (based on the matched group).

Covariate	Hazard ratio	95% Confidence interval	*P* value
Marfan	1.061	(0.248–4.544)	0.936
BMI	1.116	(0.882–1.411)	0.360
Post-descending > 30 mm*	7.804	(1.426–42.699)	0.003
Post-abdominal > 30 mm*	16.503	(2.796–97.389)	0.002

Marfan, Marfan syndrome; BMI, body mass index; Postdescending, maximal descending aortic diameter postoperatively; Postabdominal, maximal abdominal aortic diameter postoperatively.

* > 30 mm represents patients with aortic diameters above normal. The *P* value of the test of proportional hazards assumption was 0.630.

There were 16 surgical reinterventions for cardiovascular disease in 10 patients. A total of 7 MFS patients received 12 procedures, and 3 non-MFS patients received 4 surgical interventions. Overall, there were 3 patients with aortic arch reinterventions, 4 patients with descending aorta reinterventions and 2 patients with abdominal aorta reinterventions in the MFS group. In the non-MFS group, 2 patients underwent abdominal reinterventions, and 1 patient received aortic arch and descending aorta surgeries. One MFS patient underwent total arch replacement combined with frozen elephant trunk implantation due to type A aortic dissection 6.6 years after the scheduled Bentall procedure and received open repair of a thoracoabdominal aortic aneurysm 1.9 years later. In addition, MFS patients were more likely to have atrioventricular valve involvement. Two out of 10 patients in the reintervention group died. The 2 MFS patients had late death after the reinterventions caused by mitral valve dysfunction and heart failure. More detailed information is provided in Supplementary Table S2.

### 3.5. Late survival and other adverse events

**Figure 4:**
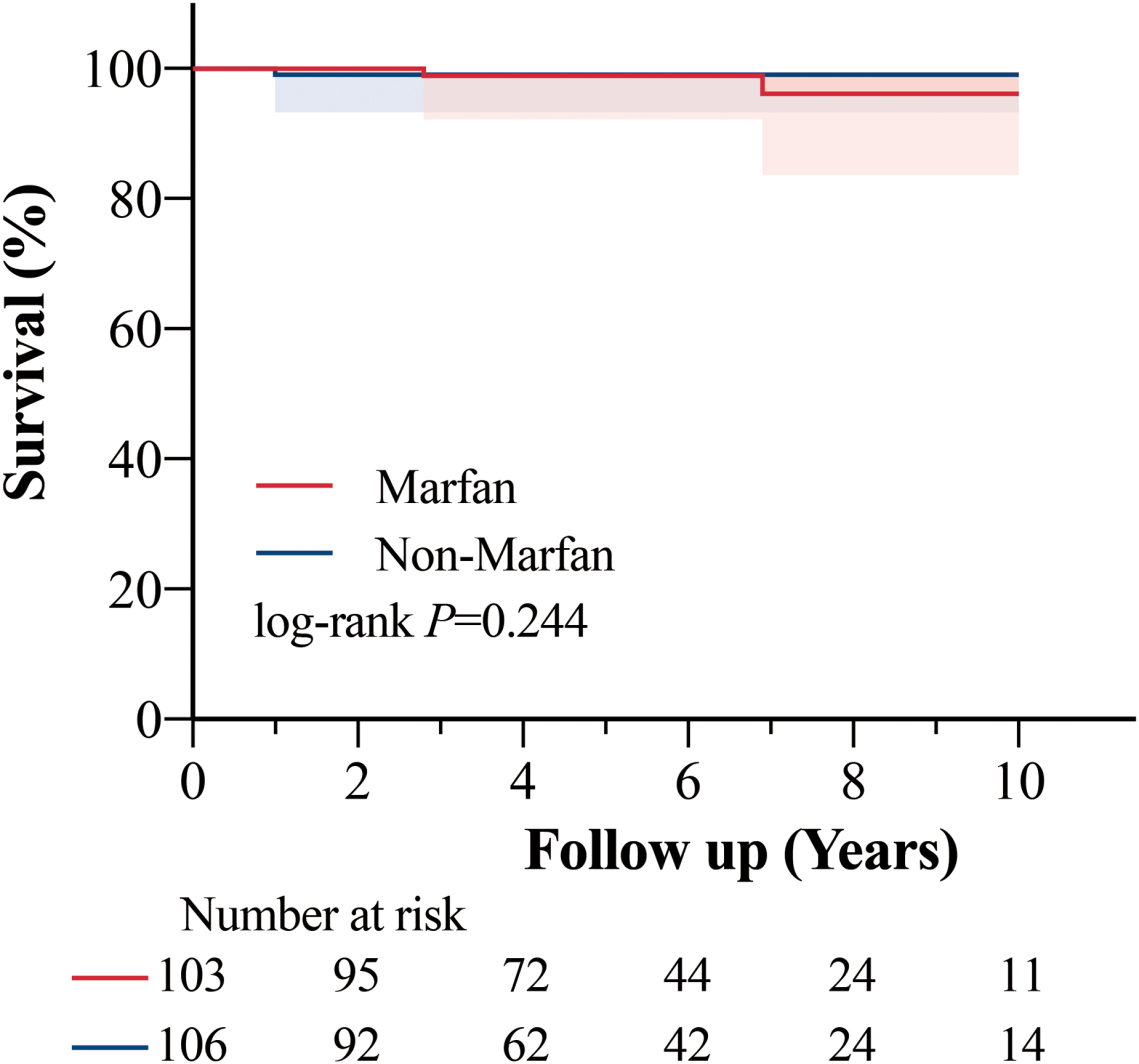
Kaplan–meier curve of all causes of death. The ten-year survival probability was 94.7% (95% confidence interval, 84.8%–98.2%) in the Marfan group and 97.3% (95% confidence interval, 91.9%–99.1%) in the non-Marfan group (*P *= 0.244). Shading represents the 95% confidence interval.

The nonaortic-related adverse events are also presented in Table 3. No difference was observed in these events. However, MFS patients had higher odds of suffering from cardiac dysfunction, although the difference did not reach statistical significance (3.8% vs. 0, *P *= .057). The overall survival at 10 years was 94.7% ± 2.9% and 97.3% ± 1.5% in the MFS and non-MFS groups (log-rank, *P *= 0.244), respectively (Figure 4). Three MFS patients and 1 non-MFS patient died during the follow-up period. The main causes of death were mitral valve dysfunction and cardiac failure in MFS patients (Supplementary Table S4).

## 4. Discussion

Most previous studies have been confined to MFS populations. No direct comparisons were made between MFS and non-MFS patients in the distal aortic segments. Schoenhoff and colleagues (8) investigated the fate of untreated distal aortas in 86 MFS patients and found that acute aortic dissection led to an increased requirement for interventions on downstream aortic segments. The same conclusion was reached in other studies (9–11, 21). It is not difficult to conclude that the fate of residual aorta in patients with aortic dissection depends on thrombosis of the false lumen and aortic remodelling (22). Therefore, the inclusion of patients with aortic dissection may have confounded the role of MFS in the downstream aorta. In a study by den Hartog and coworkers (5), prior prophylactic aortic surgery was a risk factor for type B aortic dissection in patients with MFS. The explanation given by this study is that higher pulsatile forces on the native distal aorta may occur after proximal intervention (5).

The current study included both MFS and non-MFS patients who underwent Bentall procedures with a nondissected residual distal aorta as our subjects to eliminate potential biases caused by the prophylactic surgery itself and aortic dissection. Even when these two confounding factors were removed, the baseline data in the MFS and non-MFS groups were still unbalanced due to the disease characteristics of MFS. For instance, factors such as age may be positively correlated with aortic diameter, hypertension, aortic stiffness and aortic atherosclerosis. These factors are more relevant for descending aortic aneurysm than for aortic root and ascending aortic aneurysm (23). Even in sporadic aneurysm patients, the age of onset of aneurysm between the aortic root group and the descending aortic group was significantly different (24). Therefore, propensity score matching was performed to avoid these biases. Typical pear-shaped aortic roots remained in the MFS group after matching, which suggested that matching not only balanced potential confounding factors but also preserved the aortic features of MFS in the matched groups. Differences in aortic root and ascending aortic diameters may influence the haemodynamic and pulsatile forces on the distal aorta. However, this difference disappeared after aortic root replacement surgeries and had no negative effect on the subsequent follow-up data.

A major finding of this study is that the influence of MFS-related aortopathy on the distal aorta seems less strong in the aortic root and ascending aorta. The occurrence probabilities of adverse events and reinterventions in the distal aorta were high in the MFS group, but the differences did not reach statistical significance (adverse aortic events, 10.5% vs. 4.6%, *P *= 0.106; reintervention, 6.7% vs. 2.8%, *P *= 0.210). Meanwhile, no significant difference in the cumulative incidence of distal aortic events between the MFS and non-MFS matched groups (*P *= 0.147) suggested that MFS may not affect the distal aorta as it does the aortic root and ascending aorta. In other studies, the cumulative risk for type B aortic dissection in 10 years was 19% (SE = 4%) in the prior prophylactic aortic surgery group (5). The proportion of patients experiencing distal aortic events was 8.3% in the Engelfriet study cohort (7). Although previous studies have shown that prophylactic aortic surgery is an independent risk factor for Type B dissection (5, 7), we suppose that the reason for these outcomes is that patients who require scheduled surgeries may have greater overall MFS disease severity initially compared with patients who did not require surgeries but that this was not due to the procedure itself. For MFS patients who already need aortic root surgical treatment, prophylactic aortic root surgery is warranted considering that the effect of MFS-related aortopathy on the distal aorta is minor and the low incidence of operative risk and mortality in MFS patients with scheduled aortic root surgery (25–27).

Another notable finding in our study is the positive relationship between distal aortic diameter at the initial surgery and subsequent adverse aortic events. The latest ACC/AHA guidelines for aortic disease stated that there is a lack of research to inform the risk of dilation or dissection in the native distal aorta of MFS patients (28). It only mentioned a 5.0 cm diameter threshold in the distal aorta for surgery, as is used for the aortic root. We found that a distal aortic diameter above normal is an independent predictor of aortic events. The maximum normal aortic diameter of the descending aorta and abdominal aorta was defined as 30 mm in this study (22). In other studies, den Hartog and colleagues (5) found that MFS patients with a descending aortic diameter greater than 27 mm were more likely to have type B aortic dissection. Engelfriet and associates (7) reported similar findings that previously scheduled intervention was associated with distal aorta dilation and that the diameter of the descending aorta was an independent predictor of aortic events in MFS. This might indicate a correlation between aortic diameter and MFS-related aortopathy severity. The diameter of the aortic arch and descending aorta may be more relevant to age. Even though the non-MFS group had a larger aortic arch and descending aorta than the MFS group, the differences disappeared after matching. The abdominal aortic diameter was also smaller in the MFS group before matching, but the difference reversed after matching. This may indicate that the effect of the FBN1 mutation on the aortic arch and descending aorta is modest. ﻿According to Schoenhoff and colleagues (29), the risk of reintervention of the aortic arch is low in MFS patients undergoing prophylactic root repair. However, MFS may have some impact on the abdominal aorta.

Finally, we found no significant difference in all-cause mortality between the MFS and non-MFS groups. Open surgical strategies for staged replacement of the whole aorta presented by Ikeno and colleagues (30) showed excellent long-term outcomes. Coselli and colleagues (31) also achieved promising results of open thoracoabdominal aortic aneurysm repair in MFS patients with aortic dissection. In the current study, good results were obtained in MFS patients who received interventions on the distal aorta as well. Two out of seven patients in the reintervention subgroup (3 out of 105, successfully followed-up MFS subgroup) died, but cardiac and mitral valve dysfunction remained the main cause of death in our cohort. As recently shown by Xu and colleagues (32), MFS patients may suffer from primary cardiac impairment, which has a negative effect on postoperative cardiac adverse events.

Taken together, we illustrated that the spatial heterogeneity in MFS-related aortopathy in the distal aorta is less affected than the aortic root. From another perspective, this again emphasizes the importance of preventive interventions on the proximal aorta in MFS patients with a definite indication. The evaluation of the distal aorta in MFS patients at the first surgery is important even when they present mainly with aortic root dilation. Patients with a slightly enlarged descending or abdominal aorta should continue to have imaging-based surveillance of the residual aorta after root repair. Close monitoring of cardiac and mitral valve function by echocardiogram is also required in MFS patients after aortic surgery.

## 5. Study limitations

This study is subject to the limitations inherent to single-centre retrospective observational studies. Different mutation types of FBN1 exhibit variable disease severity, and information on the mutation in patients with MFS was lacking in this study. In addition, patients could have non-syndromic hereditable thoracic aortic disease (famillial thoracic aortic aneurysm) with an identified pathogenic variant in the non-MFS group. This could explain the lack of difference between the MFS and non-MFS groups. Although there were very few patients with the family history of aneurysm (2 out of 1,200) in the non-MFS group. The diagnosis of familial thoracic aortic aneurysm can only be confirmed by genetic testing. The retrospective nature of this study did not allow for examination of genetic testing. These factors may have interference in conclusion. With a relatively low median (5 years) and interquartile range (3.1 to 7.7 years) follow-up time, the 10-year follow-up data were largely determined by a small subset of the study sample from the initial years of the study period. As such, there was a proportionally smaller cohort to evaluate long-term (10 years) outcomes. In addition, loss of patients during follow-up occurs when studies last for long time periods, and the follow-up rate was 79.2% in this study after extensive efforts. The postoperative dilation of the residual native aorta is temporal. Therefore, the distal aortic progression speed will be considered a sort of aortic dilation in future studies.

To minimize confounding factors, we utilized strict inclusion criteria and performed propensity score matching. After matching, the standardized mean differences remained high for several of the variables (e.g., aortic root Z scores) evaluated in this study. The groups are not perfectly balanced, highlighting the inherent characteristics of MFS itself. Although it retained features of MFS, the methodological limitations of propensity score matching were also presented. Loss of some population samples was inevitable due to propensity score matching. In combination with the low prevalence of distal adverse aortic events, statistical significance was not achieved, which may be attributed to the relatively small sample size. The sample size needs to be further expanded, and ongoing follow-up of this cohort is warranted.

In addition, we did not pursue a detailed comparison of different types and sizes of prosthetic aortic valves, which may impact the haemodynamic and pulsatile forces on the downstream aorta. Aortic valve-sparing root replacements were not included in this study.

## 6. Conclusions

There were no substantial differences in adverse distal aortic events and mortality between MFS and non-MFS patients after the scheduled Bentall procedure. Therefore, MFS patients who fit the indications for prophylactic aortic root surgery are highly recommended for surgical treatment. A distal aortic diameter >30 mm is an independent risk factor for aortic-related complications in MFS patients. Therefore, such MFS patients should have a comprehensive evaluation of the entire aorta at the initial procedure and receive regular imaging follow-up. Mitral valve and cardiac functions should also be evaluated regularly in MFS patients after aortic root replacements.

## Data Availability

The original contributions presented in the study are included in the article/**Supplementary Material**, further inquiries can be directed to the corresponding author.
