# Supplementary material for: Fate of the distal aorta following root replacement in Marfan syndrome: a propensity score matched study
Source: Front Cardiovasc Med. 2023 Jun 28;10:1186181. doi: 10.3389/fcvm.2023.1186181 (PMC10338094; doi:10.3389/fcvm.2023.1186181)
Supplement: Supplementary file 2 [file Table2.docx]

**Table S1** ﻿Intra-operative characteristics and postoperative outcomes

|  | **Unmatched group** | |  | **Matched group** | |  |
| --- | --- | --- | --- | --- | --- | --- |
| **Variables** | **MFS(n=226)** | **Non-MFS(n=1200)** | ***P* value** | **MFS(n=134)** | **Non-MFS(n=134)** | ***P* value** |
| Concomitant procedure |  |  |  |  |  |  |
| ﻿ Mitral valve surgery* | 61 (27.0) | 168 (14.0) | <.001 | 32 (23.9) | 26 (19.4) | 0.373 |
| Tricuspid valve surgery* | 17 (7.5) | 55 (4.6) | 0.064 | 7 (5.2) | 9 (6.7) | 0.606 |
| CABG | 5 (2.2) | 153 (12.8) | <.001 | 4 (3.0) | 3 (2.2) | >.999 |
| Tissue valve | 1 (0.4) | 111 (9.3) | <.001 | 1 (0.7) | 2 (1.5) | 0.500 |
| ﻿Cardiopulmonary bypass, min | 119 (99-143) | 126 (103-154) | 0.011 | 117.5 (96-145) | 131 (102-150) | 0.115 |
| ﻿Aortic cross-clamp, min | 82 (66-100) | 86 (68-108) | 0.035 | 82 (65-103) | 86 (69-109) | 0.206 |
| ICU stay, h | 20 (16-24.5) | 20 (17-25) | 0.299 | 20 (17-23) | 20 (16.5-24) | 0.882 |
| Mechanical ventilation time, h | 16 (11.5-19) | 17 (13-19) | 0.002 | 15 (12-18) | 16 (12-18.5) | 0.281 |
| ﻿Hospital stay, days | 15 (12-20) | 16 (13-20) | 0.517 | 15 (13-19) | 14 (12-18) | 0.106 |
| In hospital death | 2 (0.9) | 8 (0.7) | 0.664 | 2 (1.5) | 2 (1.5) | >.999 |

Values are presented in as median (interquartile range) or n (%). Marfan, Marfan syndrome; CABG, coronary artery bypass grafting; ICU, intensive care unit stay time. *Mitral valve surgery and Tricuspid valve surgery include valve repair and replacement.

**Table S2** Reinterventions in the matched groups

| **Sex** | **Age, y*** | **Marfan** | **Indication for reinintervention** | **Intervention** | **Postoperative time, y** |
| --- | --- | --- | --- | --- | --- |
| Male | 23 | Y | Type A aortic dissection | TAR + FET | 6.6 |
|  |  |  | TAAA | Open repair of TAAA | 8.5 |
| Male | 29 | Y | Type A aortic dissection | TAR + FET | 6.9 |
| Male | 30 | Y | Infective endocarditis | MVR | 4.6 |
| Female | 34 | Y | Mitral regurgitation | MVR | 6.5 |
|  |  |  | Tricuspid regurgitation | TVP | 6.5 |
| Male | 34 | Y | Mitral regurgitation | MVR | 10.6 |
|  |  |  | Tricuspid regurgitation | TVP | 10.6 |
|  |  |  | Type B aortic dissection | stented elephant trunk procedure | 10.6 |
| Male | 36 | Y | Type A aortic dissection | TAR + FET | 1.9 |
| Female | 46 | Y | Abdominal aortic aneurysm | AAAR | 2.5 |
|  |  |  |  | Common iliac artery repair | 2.5 |
| Female | 32 | N | Abdominal aortic aneurysm | AAAR | 1.7 |
|  |  |  |  | Common iliac artery repair | 1.7 |
| Male | 46 | N | Abdominal aortic aneurysm | EVAR | 2.0 |
| Male | 47 | N | Type A aortic dissection | TAR + FET | 0.4 |

Y, Yes; N, No; TAR+ FET, total arch replacement combined with frozen elephant trunk implantation; TAAA, thoracoabdominal aortic aneurysm; MVR, mitral valve replacement; TVP, tricuspid valvuloplasty; AAAR, abdominal aortic aneurysm repair; EVAR, endo-Vascular aneurysm repair. *Age at the time of Bentall procedure.

**Table S3** Univariate cox regression analysis for all aortic-related adverse events (based on the matched group)﻿

| **Variables** | **Hazard ratio** | **95% Confidence interval** | ***P* value** |
| --- | --- | --- | --- |
| Marfan | 2.154 | (0.748-6.208) | 0.155 |
| Age | 0.962 | (0.914-1.012) | 0.138 |
| Male sex | 0.685 | (0.195-2.406) | 0.555 |
| BMI | 1.254 | (1.060-1.485) | 0.009 |
| Z score | 1.030 | (0.935-1.133) | 0.550 |
| BAV | 0.318 | (0.042-2.412) | 0.268 |
| Hypertension | 0.798 | (0.227-2.807) | 0.726 |
| CHD | 3.213 | (0.419-24.621) | 0.261 |
| Post-arch, mm | 0.947 | (0.816-1.098) | 0.470 |
| Post-descending, mm | 1.150 | (1.053-1.257) | 0.002 |
| Post-abdominal, mm | 1.226 | (1.070-1.404) | 0.003 |
| Post-descending  > 30 mm* | 3.905 | (0.861-17.721) | 0.077 |
| Post-abdominal  > 30 mm* | 10.539 | (2.112-52.591) | 0.004 |
| Propensity score | 1.306 | (0.224-7.598) | 0.767 |

Marfan, Marfan syndrome; BMI, Body mass index; BAV, Bicuspid aortic valve; CHD, coronary heart disease; Post-arch, descending, abdominal, maximal aortic arch, postoperative descending and abdominal aortic diameter. *****>30 mm represents patients with aortic diameter above normal.

**Table S4** ﻿Death during follow-up in the matched groups

| **Postoperative time, m** | **Sex** | **Age, y*** | **Marfan** | **Cause of Death** |
| --- | --- | --- | --- | --- |
| 0 | Male | 34 | N | ventricular wall motion abnormality, poor cardiac function |
| 0 | Female | 52 | Y | refractory postoperative infection |
| 0 | Female | 63 | Y | ventricular fibrillation during hemostasis, poor left ventricular function, unstable blood pressure under ECMO support |
| 0 | Female | 55 | N | ventricular fibrillation, poor cardiac function |
| 12 | Male | 71 | N | sudden death at home (cause of death is unknown) |
| 34 | Male | 34 | Y | heart failure |
| 83 | Female | 34 | Y | mitral valve dysfunction, heart failure |
| 140 | Male | 34 | Y | mitral valve dysfunction, heart failure |

Y, Yes; N, No; ECMO, extracorporeal membrane oxygenation. *Age at the time of Bentall procedure.
