# Supplementary figures and images for: Fate of the distal aorta following root replacement in Marfan syndrome: a propensity score matched study
Source: Front Cardiovasc Med. 2023 Jun 28;10:1186181. doi: 10.3389/fcvm.2023.1186181 (PMC10338094; doi:10.3389/fcvm.2023.1186181)

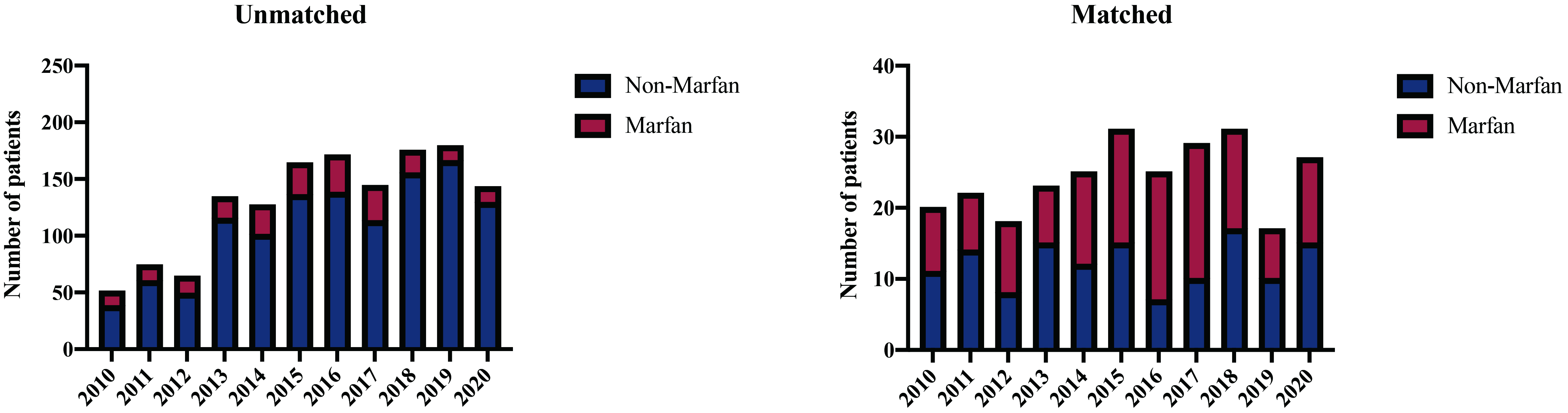

Supplement: Supplementary file 3 [file Image1.tif]

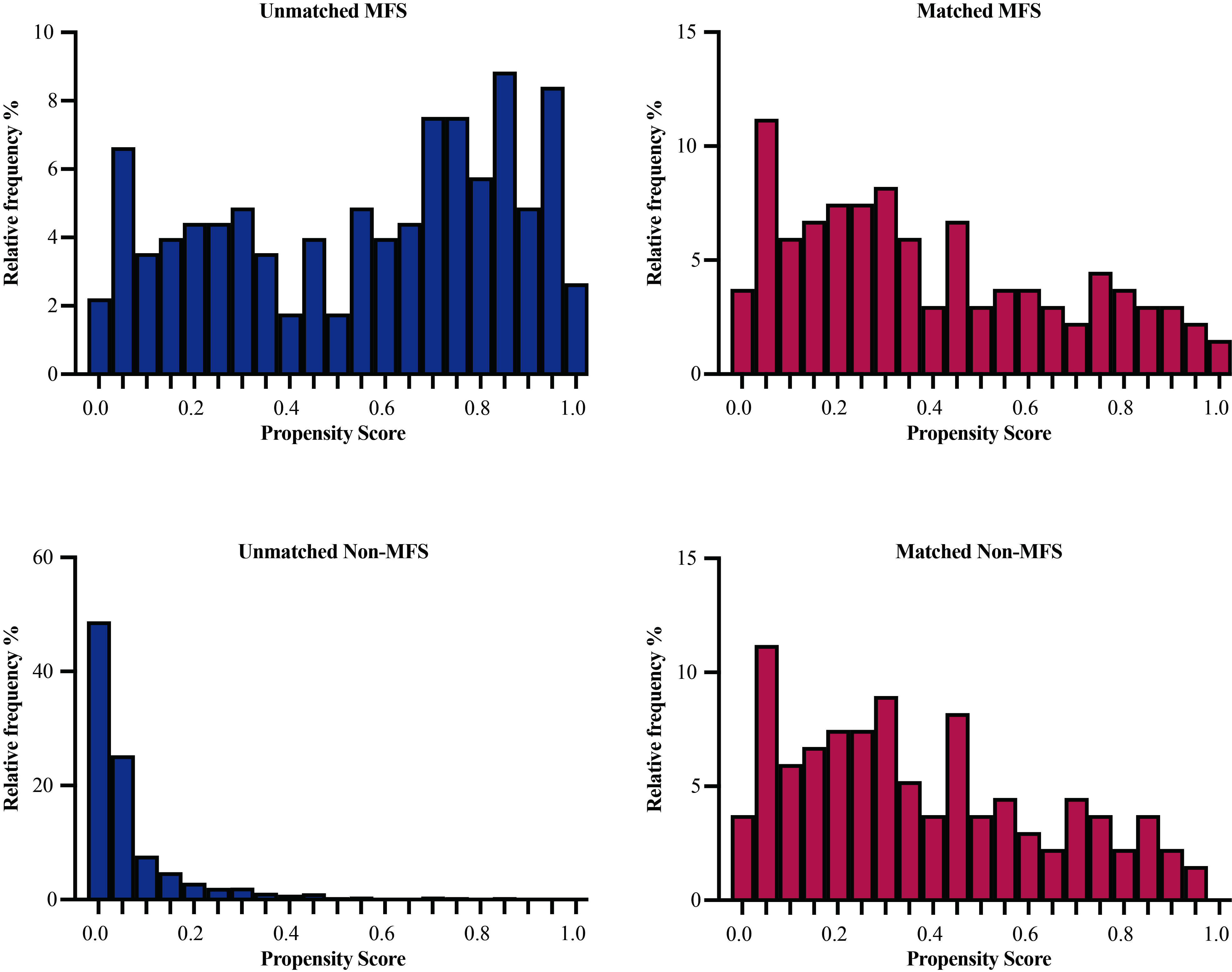

Supplement: Supplementary file 4 [file Image2.tif]

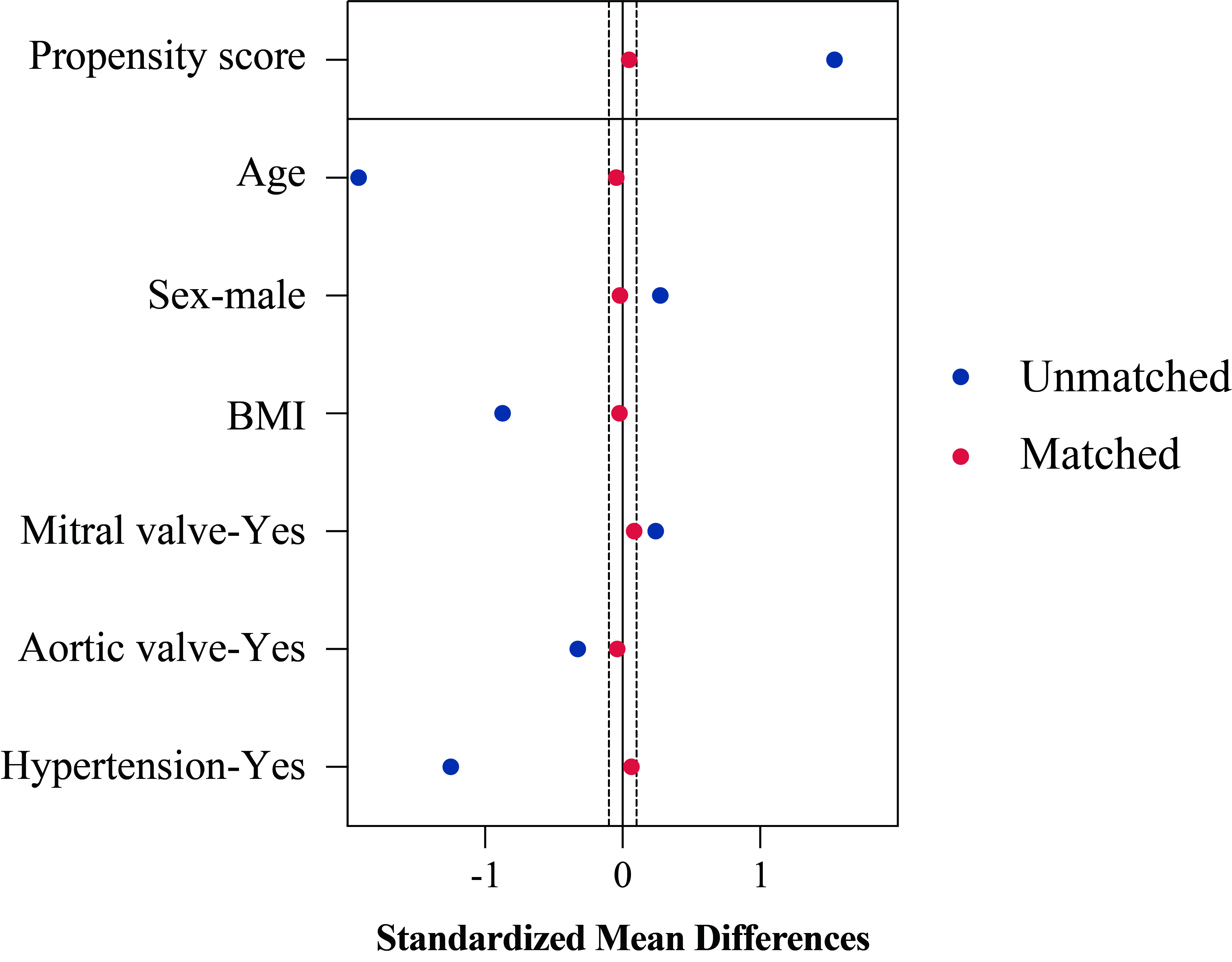

Supplement: Supplementary file 5 [file Image3.tif]

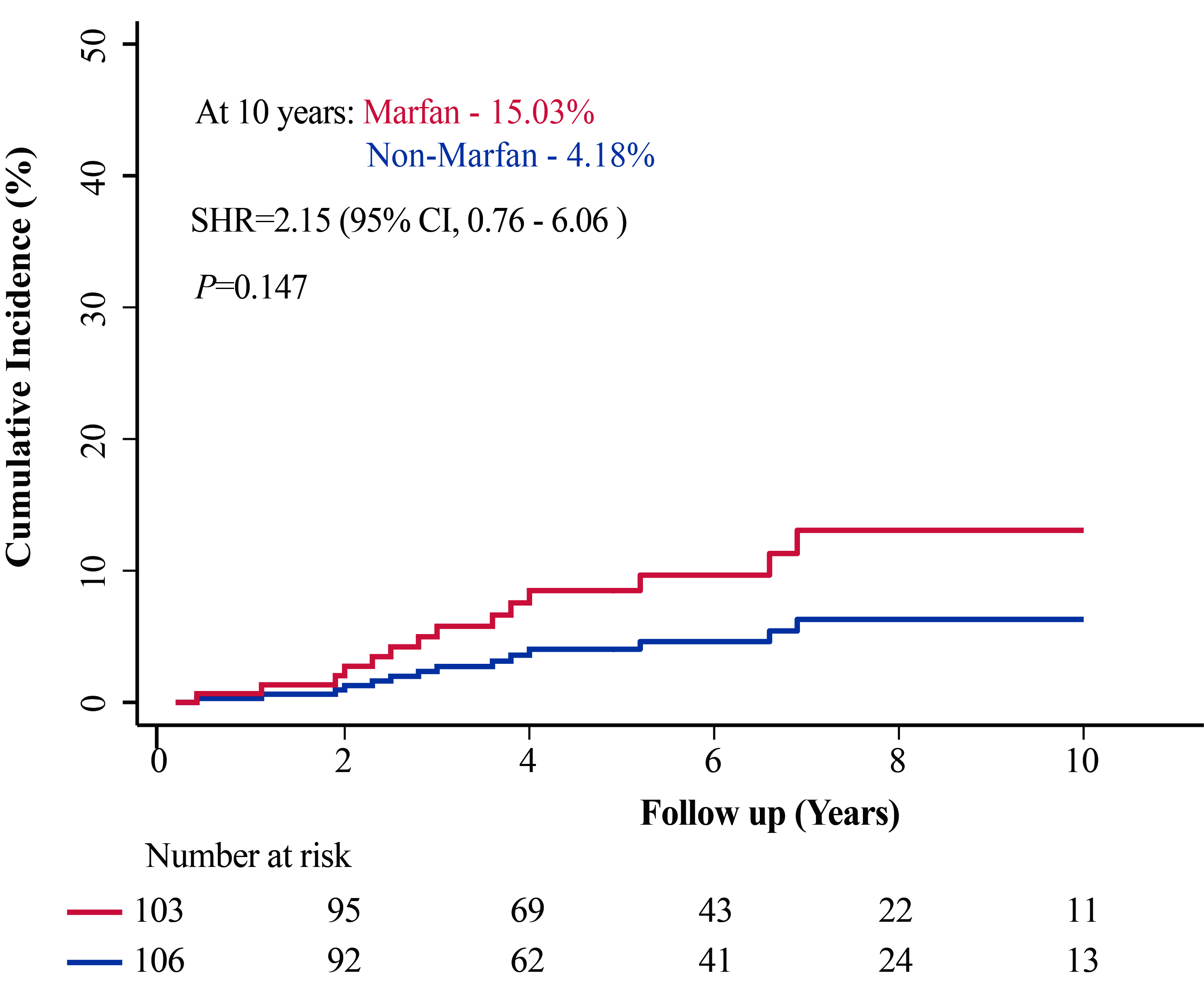

Supplement: Supplementary file 6 [file Image4.tif]
